# Using participatory methods to develop a narrative intervention to alleviate distress in children hospitalised with TB in South Africa: The DIMPle project

**DOI:** 10.1371/journal.pone.0338394

**Published:** 2025-12-10

**Authors:** Caitlin D. October, Dzunisani P. Baloyi, Lario Viljoen, Rene Raad, Dillon T. Wademan, Megan Palmer, Juli Switala, Michaile G. Anthony, Karen Du Preez, Petra De Koker, Anneke C. Hesseling, Bronwyne Coetzee, Graeme Hoddinott

**Affiliations:** 1 Desmond Tutu TB Centre, Department of Paediatrics and Child Health, Faculty of Medicine and Health Sciences, Stellenbosch University, Cape Town, South Africa; 2 Department of Global Health and Development, Faculty of Public Health and Policy, London School of Hygiene and Tropical Medicine, London, United Kingdom; 3 The Aurum Institute, Johannesburg, South Africa; 4 Department of Psychology, Stellenbosch University, Cape Town, South Africa; 5 School of Public Health, Faculty of Medicine and Health, The University of Sydney, Sydney, New South Wales, Australia; Georgetown University Medical Center, UNITED STATES OF AMERICA

## Abstract

Children who are hospitalised for tuberculosis (TB) experience challenges that put them at risk of developing emotional, behavioural, and social difficulties. In this methodological paper, we showcase the development of a narrative intervention toolkit with key components of the resulting version 1.0 tool. The study design was participatory and pragmatic, with researchers working with the routine staff of TB hospital wards, children admitted and their caregivers, to iteratively understand and improve children’s experiences of hospitalisation. The project included three phases: (1) a situational analysis to map children and healthcare providers’ perspectives on priorities and potential intervention components, (2) co-development of a beta-version of the intervention, and (3) piloting and incremental refinement toward a version 1.0 of the intervention. The intervention toolkit combined a series of activities alongside the story of ‘Courageous Curly’ to facilitate children’s engagement with their own experiences of hospitalisation, including psychosocial and treatment challenges, captured, and described throughout data collection. We found that dividing the story into short chapters facilitated children’s engagement with the section of story that is being told on a specific day. Each chapter of the story follows/mimics a different stage children can expect during their treatment journey while hospitalised for TB care. Implementation and evaluation of such interventions can mitigate the psychosocial impact of TB in children and inform policies to improve their overall TB care.

## Background

Globally, an estimated 1.3 million children were diagnosed with tuberculosis (TB) in 2022 [1]. In 2022, 15 000 children <15 years are estimated to have developed TB in South Africa [2]. The World Health Organization (WHO)’s guidelines for the treatment of drug-susceptible TB currently recommend a 4–6 months course of anti-TB drugs [1], while for children receiving treatment for rifampicin/multidrug-resistant (RR/MDR-TB) the recommendation remains 9–18 months. Historically, patients undergoing RR/MDR-TB treatment were hospitalised for up to 18 months due to the daily injectable agents that had to be administered by nurses [3]. Due to significant adverse effects and need for admission there has been a shift towards injectable-sparing regimens [4] and community-based care for people with RR/MDR-TB.

In 2018, MDR-TB treatment for children moved away from using daily painful injectable medicines which required nurse administration and often, months of hospital-based care [5]. However, in many high RR/MDR-TB burden settings around the world, children’s treatment still includes a period of hospitalisation [6,7]. The main reasons are due to the complexity of regimens and monitoring and limited access to decentralised care [8]. Children admitted with drug-susceptible TB (DS-TB) are usually admitted due to significant illness, severe disease (TB meningitis), human immunodeficiency virus (HIV) co-infection, requiring more complicated regimens or significant social care concerns. As such these children have experienced significant trauma prior to admission. Children who are hospitalised for TB treatment are often from vulnerable families with disruptive social/ home environments [6]. Inconducive and challenging home environments mean that caregivers either have financial strain or are physically unable to care for their children [9].

Adverse effects experienced during hospitalisation in children with MDR-TB include bodily pain, feeling nauseous, fear of medical procedures and concern about the stigma associated with being hospitalised for MDR-TB [10–15]. Socially, many children with TB, especially MDR-TB, experience isolation [15], negative impacts on mental well-being [6], and economic hardships [16]. These challenges may be experienced directly or vicariously through their household/ caregiving unit. As a result of these challenges, children who are hospitalised for long periods with MDR-TB are at risk of developing emotional, behavioural, and social difficulties which contribute to the stressful experience of hospitalisation itself [6,13].

Apart from physical effects, extended hospitalisation in childhood is associated with psychological and social disruption [17–19]. In a study in Turkey, Boztepe and others (2017) found that children 6–12-years of age who were hospitalised for various illnesses were at increased risk of developing anxiety due to being in an unfamiliar environment and being ill-informed about medical procedures [18]. Children in this study also missed their friends from school and missed out on educational time. Obaid (2015) interviewed mothers of children hospitalised for cancer treatment/care and found that the children displayed negative mood changes, bad tempers, and a loss of appetite due to being in an unfamiliar environment. Similarly, in a study in a rural setting in the United States of America with paediatric oncology patients 7–18-years-old who had been hospitalised for at least three nights, found that children feel isolated and lonely [19].

The WHO global strategy for TB research and innovation (2020) stipulates that to achieve patient-centred care, one of the three pillars of the WHO’s End TB Strategy [20], evidence-based approaches need to be used to determine how best to adapt and implement global recommendations on interventions for TB care [21]. It further notes that social science research can be used to identify how context-specific performance gaps may be closed. A final recommendation relevant to this research is that new interventions should be assessed in terms of their feasibility, acceptability, effectiveness and impact on health outcomes and healthcare systems [21].

We report on a continuation of the DIMPLe Project (Developing simple, cost-efficient Interventions to Manage Psychosocial distress associated with Long-term hospitalisation in children affected by TB), with formative work described by Meyerson and colleagues (2021) and Baloyi and colleagues (under review) [22]. Baloyi and colleagues (under review) found that interventions to address the negative consequences of hospitalisation for children with TB typically include clown therapy, music therapy, various forms of play, animal-assisted interventions, drawing, journaling, and cognitive-behavioural interventions. In this methodological paper, we showcase the development of a narrative intervention toolkit with key components of the resulting version 1.0 tool. In a future study, we will examine the effectiveness of the intervention toolkit.

## Methods

### Setting

Cape Town is a high TB-burden setting in South Africa with approximately 730 cases of TB per 100 000 population [23]. There were approximately 29 000 cases of TB in Cape Town in 2023 [24]. Most TB services are delivered through a network of >100 primary health care facilities (‘clinics’) across the city. Brooklyn Chest Hospital (BCH) is a regional specialist TB hospital for all people who require in-patient care for TB treatment. Although caregivers are allowed to sleep at the hospital, this occurs rarely, due to insufficient space, concerns about infection control if mothers have undiagnosed TB themselves, maternal responsibilities and sometimes being referred long distances for admission having socio-economic constraints. Some children spend weekends at home if their families can support this. Children’s admission and discharge from BCH is dynamic and somewhat unpredictable. Since many children spend many months in hospital, schooling is provided in the form of an early learning centre or ‘creche’ for 0–5-year-olds with two carers, and an all-age mixed class for >5-year-olds facilitated by one educator, between 09:00–14:00 on weekdays.

### Design

The study design was participatory and pragmatic, with researchers working with the routine personnel of the hospital wards, children at the TB hospital and their caregivers to iteratively improve children’s experiences of hospitalisation. Participatory studies focus on bringing about change and are practical and collaborative as the research incorporates inputs from the participants that the change is aimed at [25].

The project included three phases: (1) a situational analysis to map children and healthcare providers’ perspectives on priorities and potential intervention components, (2) co-development of a beta-version of the intervention, and (3) piloting and incremental refinement toward a version 1.0 of the intervention.

#### Phase 1: Situational analysis.

***Sample*:** A convenience sample of all children (6–13-years-old; n = 4) receiving in-hospital care between September and November 2021. This included children with both DS-TB and RR/MDR-TB and children with both pulmonary and extrapulmonary TB. We also recruited caregivers of children (<6-years-old; n = 4) receiving in-hospital care in the same period, and their healthcare providers; a doctor, two nurses, and two educators who manage the school.

***Data collection*:** We conducted 7 full days of observations in the children’s ward at BCH over the month of August 2021 which involved following children’s processes through admission, care, and treatment at the hospital. The first author (CDO) observed the interactions between the children and challenges they faced during hospitalisation. During the observations, children often cried and displayed signs of aggression. For example, one of the girls in the creche at BCH shoved and kicked the other children.

We then conducted in-depth qualitative interviews with children, their caregivers and staff between September and November 2021 which supplemented the observations that were conducted in August. Each interview was audio-recorded and lasted for 30–45 minutes. A semi-structured interview guide was followed with topic areas on getting to know the participants, the challenges that the children experience during hospitalisation and suggested solutions to these challenges. The data collectors (CDO and a graduate research assistant trained in psychology) kept detailed notes on a standard case report form.

***Data analysis*:** We organised participants’ reported intervention priorities thematically with between case comparisons. All emergent ideas were discussed with experienced socio-behavioural scientists BC, GH and LV.

#### Phase 2: Co-development of a ‘beta’ version of the intervention.

***Sample*:** We enrolled a convenience sample of health workers (n = 5) (including a doctor, nurses and educators) working in the children’s ward. We asked the health workers not occupied and those who engaged with the children daily (n = 5) to both provide input on the intervention.

***Data collection*:** While the narrative intervention toolkit was being drafted between March 2022 and September 2022, CDO took drafts of the intervention to health workers for their input on the intervention while it was being developed. Notes were made based on health workers’ feedback and integrated into the intervention toolkit.

#### Phase 3: Piloting and incremental refinement of the intervention.

***Sample*:** We enrolled a convenience sample of the children between the ages of (6–12-years-old; n = 6) attending the hospital school. This sample also included children with both DS-TB and RR/MDR-TB and children with both pulmonary and extrapulmonary TB.

***Data collection*:** The first draft of intervention toolkit, “*Courageous Curly”,* was piloted in the school at the hospital in September 2022 and observed by CDO. Courageous Curly was implemented by the hospital schoolteacher. The hospital schoolteacher was also actively involved in the development process during the preceding phases. One session of the implementation of the intervention was held each day over 13 days, each lasting approximately 45 minutes. CDO was present to observe each session. The lessons learned, as seen in Table 2, were drawn directly from these observations. We conducted naturalistic observations, which involved observing the participants in their natural environment [25], at the hospital’s school to witness the children’s interactions with Courageous Curly. CDO recorded children’s interactions with the intervention toolkit through written notes following a structured observation guide. CDO then interviewed hospital staff about using the intervention toolkit and children about their experiences of taking part in the intervention toolkit.

**Table 2:** Lessons learnt.

Lesson	Illustrative example	Recommendation
The intervention can be successfully integrated into in-hospital routines for children aged 6–13.	The weekly teaching routine included daily classes between 09:00–14:00 on weekdays. We offered a 30–45 minute session that was incorporated into the daily classes	For the implementation of an in-hospital intervention, intervention sessions of 30–45 minutes should be incorporated into in-hospital routines
Collaboration with health workers, educators, and parents is essential for buy-in.	This iterative engagement helped ensure that version 1.0 of the toolkit was shaped by those directly involved in the children’s care, enhancing its practicality and acceptability in the hospital setting.	The development of an intervention for hospitalised children should be an iterative process with those directly involved with the children’s care
Including events and experiences children can relate to enhances engagement.	Each chapter of the story follows a different stage children can expect during their hospital journey – such as admission, taking medication, and making friends – which helped them relate their own experiences to those in the intervention	A narrative intervention should depict various experiences that the targeted audience can relate to
Breaking the story into short parts helps maintain children’s attention.	Health workers recommended that the story be broken into short segments told over several days. In response, the story was divided into four chapters, each with three sections presented across 13 days, allowing children with different ages, language abilities, and attention levels to stay engaged.	Stories for children should be broken into short parts to maintain their attention
Interactive activities are useful for eliciting children’s own narratives.	Children were prompted to interact with Curly by sharing their own stories. They engaged in drawing, playacting, and writing letters, which allowed them to express their own hospital experiences.	Narrative interventions should include interactive activities, such as play and drawing, so that children have an avenue to express their own narratives
Observations help identify relevant events to include in the story.	The emotional and social dynamics (also described in Meyerson et al, 2021) that were observed to inform the need for developing the intervention were translated into the story’s events	A narrative intervention should depict the dynamics between the targeted audience so that they can relate to events in the story
Sharing drafts with health workers and integrating their feedback improved the toolkit’s relevance and usability.	While drafting the toolkit, health workers who interacted daily with the children were consulted regularly. One suggestion was to use familiar animals and Xhosa names to make the story more relatable. These and other recommendations were integrated into the final version of the intervention, ensuring cultural and contextual relevance	Those who will implement the narrative intervention should be included in the drafting phases of the intervention to reach maximum usability of the intervention

Courageous Curly was presented over 13 days to children hospitalised at BCH. Over the 13 days of the presentation of the intervention, a combined total of six children were observed. One child was discharged on the fourth day of intervention toolkit presentation. Another child who partook in the intervention was discharged prior to the interviews which took place three months after intervention completion. The final sample of children (n = 6) who were interviewed about their experiences had all participated in Courageous Curly at the hospital school. Two children that had participated in the interviews had taken part in presentations of the plays after the intervention toolkit had been presented. The interviews were conducted in the children’s preferred language (English or Afrikaans) by CDO who is fluent in English and well-versed in Afrikaans.

***Data analysis:*** Interviews were translated and translations were checked by multiple bilingual individuals to ensure accuracy. We used Braun and Clarke’s (2021) six phase reflexive thematic analysis process to analyse the data. These phases are as follows: Familiarisation; coding; generating initial themes; reviewing themes; defining and naming themes; writing up [26]. Themes reflected children’s experiences of being in hospital after they had heard the story.

### Ethical considerations

The project was approved by the Health Research Ethics Committee of Stellenbosch University; Ethics Reference No: N19/04/053. The management team of the children’s ward at BCH provided approval for this study to take place. Staff suggested children who could participate, and parents were contacted and provided with consent forms in their preferred language, which were read aloud when necessary. Parents were reassured that the child’s willingness to participate would be confirmed at every interaction, and that any data collected would be removed if the child chose to withdraw. For children, assent was obtained with the schoolteacher present. All children who participated in the narrative intervention had provided consent before implementation, and no incentives were provided that could have coerced participation. In the consent form for the children and health workers to partake in first phase of interviews and observations for the development of the intervention toolkit, the stated protocol aim was to work with children and adolescents, caregivers, and health workers using participatory research to design an intervention to better manage psychosocial distress during TB treatment.

For phase three of this study, parents of children were contacted for their consent for their children to participate in this study. Informed consent/assent was also obtained from the children willing to take part in the interviews in phase three [27]. Informed consent was obtained from all members of staff and caregivers that were part of the interview processes. Child and health worker participants did not receive monetary reimbursement because their participation in the activities formed part of the day-to-day routine of life in the paediatric ward at the BCH. Child safeguarding protocols were in place throughout data collection. The school teacher was present to support children in case of emotional responses, and all sessions were conducted on-site where each child’s physician was readily accessible.

## Findings

### The intervention toolkit

The intervention toolkit combined a series of activities alongside the story of ‘Courageous Curly’, a caterpillar hospitalised for MDR-TB care, to facilitate children’s engagement with their own experiences of hospitalisation. During his hospitalisation, Courageous Curly has experiences that mirror those of the children. Using animals that the children see every day, such as birds and insects in the narrative was encouraged by health workers during phase two of the study. Each of the characters in Courageous Curly have local and culturally appropriate names and are animals that the children easily recognise.

**Fig 1:**
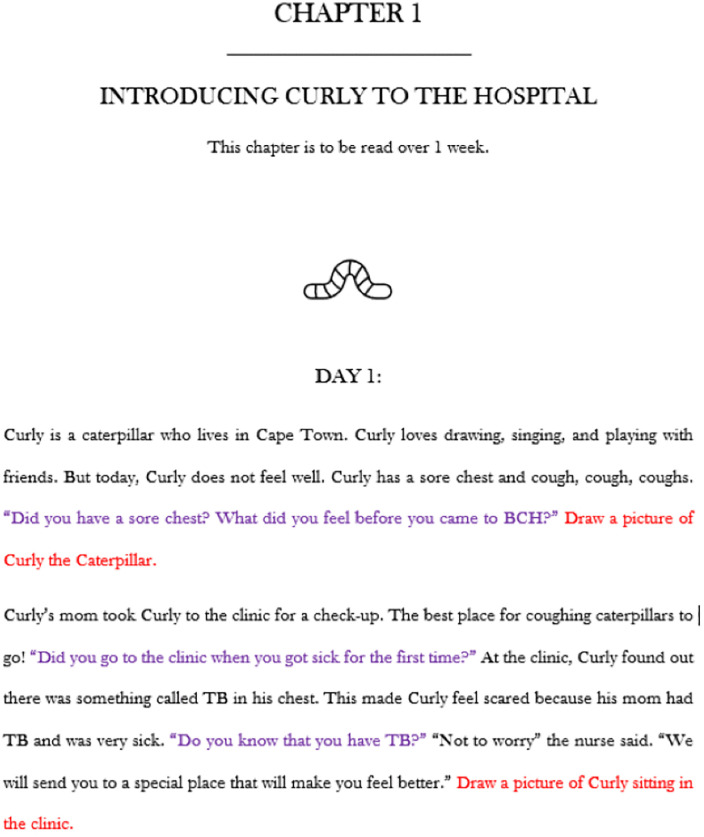
Chapter 1 of Courageous Curly.

The intervention toolkit comprises various activities differentiated by colour prompts (Fig 1). Purple prompts encourage children to interact with Courageous Curly. In these prompts, the reader of the story asks the children a question after a part of the story has been told. For example, after the reader tells the children that Curly the caterpillar made friends, the children are asked to tell Curly about the friends they have made while at hospital. Prompts in red text encouraged children to partake in activities such as draw pictures or present plays of what is happening to Curly the caterpillar. This was informed by the scoping review in which we found that strategies including play or drawing are effective at alleviating children’s anxiety associated with hospitalisation [22].

Each chapter of the story follows/mimics a different stage children can expect during their treatment journey while hospitalised for TB care. The four chapters represent four overarching sections (Table 1): (1) Introducing Curly to the hospital. (2) Who is Curly going to meet at the hospital? (3) What happens while Curly is at the hospital? and (4) Curly gets to go home! The intervention toolkit is accompanied by an instruction manual which contains guidelines for the reader to understand how the storybook is structured and how to present the story to the children.

**Table 1:** Outline of chapters in the story of Courageous Curly.

Section Chapter	1	2	3	4	5
Chapter 1: Introducing Curly to the hospital	Curly Caterpillar arrives at the hospital because he has TB – day 1	Curly sees new animals – day 2	Curly goes with the nurse to the doctor’s room – day 3	What happens to Curly in the doctor’s room? – day 4	Curly feels side effects of the TB medication – day 5
Chapter 2: Who is Curly going to meet at the hospital?	Curly plays outside with new animals – day 6	Curly meets differently abled animals – day 7	Curly meets an animal that speaks a different language – day 8		
Chapter 3: What happens while Curly is at the hospital?	Curly sees two animals having a disagreement – day 9	Curly finds out that he can write letters to his granny – day 10	All animals write letters to their families – day 11	It’s Curly’s birthday! – day 12	
Chapter 4: Curly gets to go home!	Curly feels much better – day 13	Curly says thank you to the staff – day 14	Curly has a goodbye party – day 15		

### Chapter 1: Introducing Curly to the hospital

**Fig 2:**
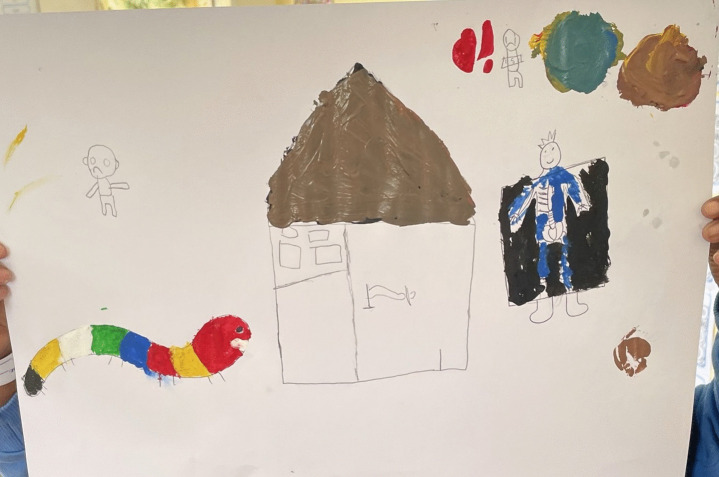
Drawing of Courageous Curly at the hospital.

In section one of chapter one, Curly is introduced to children as a caterpillar that does not feel well. The health worker or educator that is presenting the story asks the children questions about their own experiences of being diagnosed with TB before they were admitted to the TB hospital. The children are also prompted to draw pictures of Curly and what happens to him. An example of this can be found in Fig 2.

This section of the story proceeds to explain how Curly was diagnosed and admitted to hospital. The children are then prompted to tell Curly about how they felt when they first arrived at the hospital.

In section two of chapter one, the health worker or educator that is presenting the intervention reminds the children that Curly was hospitalised on day one. On day two, Curly sees there are other animals admitted to the TB hospital that also have TB. The children are prompted to talk about the friends that they have at the hospital and draw pictures of them.

**Fig 3:**
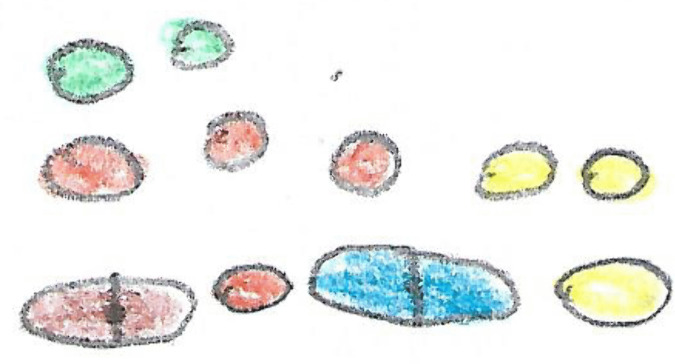
Drawing of tablets (eight-year-old girl).

In the following sections of chapter one, Curly is introduced to Doctor Owl, has his blood drawn in the doctor’s room and is given his medication. This an example of an experience that was taken from the data in phase 1, as one of the children who were interviewed said, “I was scared because I was scared to be injected with a big injection and you know it is big”. The children are asked questions about their experiences with the doctor at the TB hospital. They are also prompted to draw pictures of this experience and tell Curly about how they felt in the doctor’s room. This makes them aware that they are not alone in their experiences They are prompted to put on a play of what happens in the doctor’s room and draw pictures of the medication that they take, see Fig 3. The children listening to Curly’s story also gain knowledge about the possible side effects of their medication, but that they will feel better if they continue to take their treatment.

#### Purpose of Chapter 1.

The purpose of this chapter is to help children understand why they have been hospitalised.

#### List of activities.

- Answering questions about their experiences of being admitted to the hospital.
- Answering questions about their experiences of being in the hospital.
- Answering questions about their experiences of taking medication.
- Drawing pictures of the abovementioned experiences.
- Put on a play of what happens in the doctor’s room.

#### Intended outcomes.

After the children have heard and participated in this chapter of the story, they should be aware of why they have been admitted to the hospital and why they take medication while they are there. They should also be aware that they are not alone in their experiences of being in an unfamiliar environment with unfamiliar people and negative experiences of medical procedures that occur while they are in the hospital. It also facilitates educating children about possible side-effects of treatment and encourages them that it will help them become healthy.

### Chapter 2: Who is Curly going to meet at the hospital?

**Fig 4:**
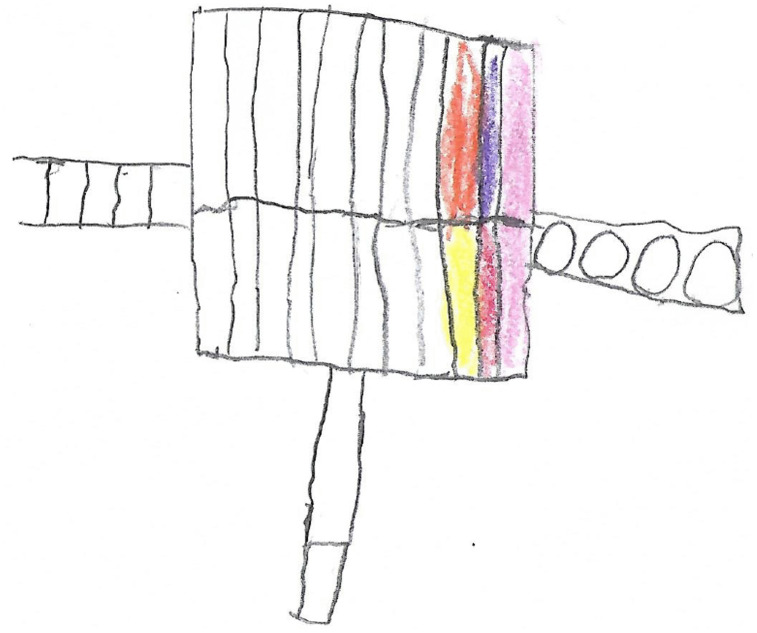
Drawing of a jungle gym (seven-year-old boy).

The presenter starts this chapter by asking the children to reflect on what happened in the story in the previous chapter. The presenter then moves on to tell the children that Curly has woken up on a new day and feels slightly better after taking his medication. On this day, Curly is feeling well enough to play outside with the other animals and he joins them playing on the jungle gym. The children are prompted to draw a picture of the jungle gym (Fig 4).

In the following sections of chapter two, Curly comes across animals with different physical appearances due to having TB. This had been added to the story as different physical appearances were noted during the observations. In the story, Nurse Nelly helps explain to Curly why the animals look different. Curly also meets a Xhosa speaking Beetle called Bongi who he becomes friends with despite their differences in languages. At the end of this section, the children are encouraged to put on a play that shows Curly how they can be friends with each other despite the differences in appearance and languages that they speak.

#### Purpose.

In this chapter, the characters in the story make friends with each other and play together. The play activities in this chapter support the children making friends as they are prompted to ask each other questions to learn about each other. Some of the characters in the story have different physical appearances due to their TB and the reasons for this are explained in the story. The purpose of this is for the children to feel more comfortable with each other despite their differences.

#### List of activities.

- Answering questions about their experiences of encountering other children at the hospital.
- Answering questions about their experiences of making friends at the hospital.
- Drawing pictures of the abovementioned experiences.
- Put on a play of how they would make friends at the hospital.

#### Intended outcomes.

Children will be made aware of the diverse population of children that are admitted to the hospital. The activities in this chapter should encourage children to become friends with each other despite their differences.

### Chapter 3: What happens while Curly is at the hospital?

The presenter of the intervention begins this chapter by reminding the children of what happened in the previous chapters. These reminders are also useful for newly admitted children so that they become familiar with the story. In section one of chapter three, conflict among characters takes place. Nurse Nelly intervenes and helps them to resolve their conflict. The children are prompted to put on a play of what happened in this section of the story and show how it was resolved.

Section two of chapter three shows the children that they can call their families at home if their families have access to phones. The children are asked to talk about whether they call their families or not. In section three of chapter three, Curly writes a letter to his own granny. The children participating in the intervention are asked to draw pictures and write letters to their families about what happens to them and Curly at the hospital. These activities are included as many children talked about missing families in phase 1. For example, a 13-year-old girl said, “Before I had phone I used to always cry, stay the whole night feeling upset and missing my mother... I miss my mother so much”. In section four of chapter three, it is Curly’s birthday. The children are prompted to organise a party for Curly and draw a picture of him enjoying his party.

#### Purpose.

The purpose of the resolution of conflict in this chapter is to show children that they have support from the staff at the hospital when they need it. This chapter also shows children that there are exciting things that happen at the hospital, such as parties.

#### List of activities.

- Talk about a time that the nurses had helped them when they needed it.
- Put on a play of how the nurses resolve conflict among the children.
- Write letters or draw pictures to show their families what they experience at the hospital.
- Draw pictures of making phone calls.
- Talk about how they feel about missing their families at home.
- Plan a party for Curly the caterpillar.

#### Intended outcomes.

Children are told that they have a network of support (hospital staff and other children) at the hospital. Children are made aware that they can call their caregivers while they are in the hospital or write letters to them if they are unable to make phone calls. Children also learn that there are events that take place at the hospital that they can look forward to.

### Chapter 4: Curly gets to go home!

**Fig 5:**
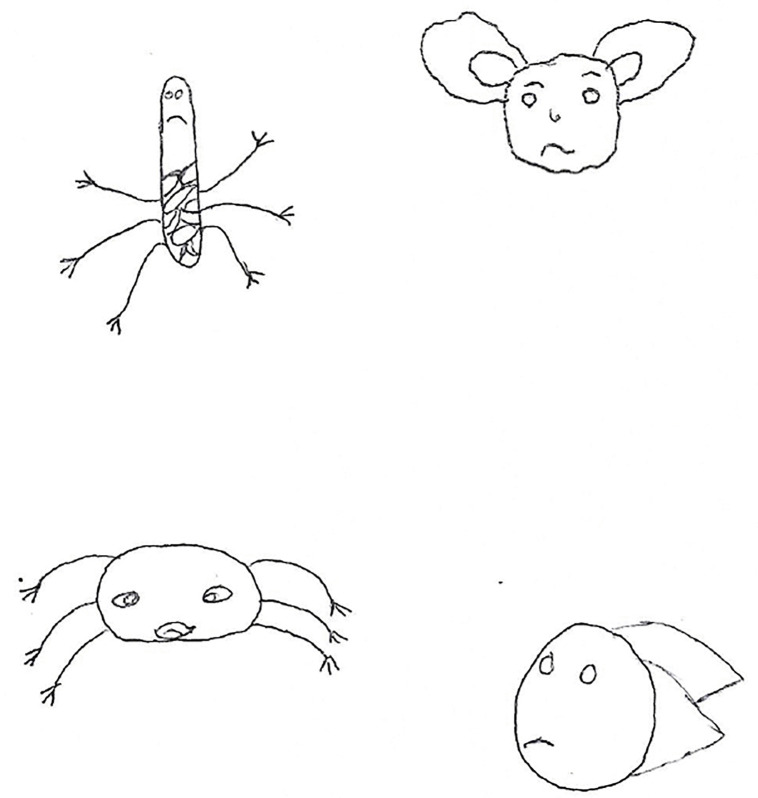
Drawing of the animals with sad faces (twelve-year-old girl).

The presenter of the intervention begins this chapter by recapping what happened in the story in the previous week. In the first section of chapter four, Curly goes to the doctor’s room where he is told that he is doing well enough to go home from the hospital. The children are asked if they are going to miss Curly and prompted to draw pictures of the animals at the hospital with sad faces (Fig 5).

In this section, Curly asks his friends at the hospital to help him with a play at his party to tell everyone his story of being at the hospital. The children are then prompted to practice a play re-enacting the part of the story where there had been conflict. The second section of this chapter is where Curly talks about the friends that he made at the hospital and says goodbye to them. Curly has his farewell party and goes home in the third and final section of the story. He thanks the hospital staff for taking care of him and leaves the hospital with his mother. The children are prompted to reflect on their favourite moments of being at the TB hospital.

#### Purpose.

Children who are about to be discharged from the hospital may experience anxiety and uncertainty and may need guidance for when they go back to their home environment. In this chapter, Curly the caterpillar is healed of his TB and is discharged from the hospital. The purpose of this chapter is for the children to understand that there is an end to their time at the hospital. The activities in this chapter allow children to say goodbye to one another and thank the hospital staff. It also allows for the children’s time spent at the hospital and being healed to be celebrated.

#### List of activities.

- Children are prompted to talk about how being at the hospital has made them feel less ill.
- Talk about things that they enjoyed or did not enjoy while being at the hospital.
- Talk about what they like about each other.
- Talk about how they will be when they get to go home to their families.

#### Intended outcomes.

This chapter allows children to reflect and take note of the positive impact that being at the hospital has had on them. Children will be shown that there is an end point of being at the hospital and they can be excited to reunite with their families and go home.

### Lessons learnt

The development and pilot implementation of version 1.0 of the intervention provided valuable lessons (Table 2) that can inform the implementation of similar interventions in similar settings. Lessons learnt include the finding that this kind of intervention can be successfully integrated into the in-hospital routine of care and education for patients aged 6–13. The intervention can be implemented by nurses or lay counsellors who do not have prior narrative intervention training, which also allows for broader implementation in hospital settings that may not have a school program. Achieving buy-in for a narrative intervention in a hospital setting requires a collaborative effort involving health workers, educators, and parents. Observations are also crucial for understanding the types of events and experiences that can be added to the story to ensure it resonates with the children. It is important to incorporate events and experiences that children can relate to, as this helps enhance engagement and relevance. Sharing drafts of the intervention with health workers during development allowed for ongoing, practical feedback, which was carefully documented and incorporated into the final toolkit to ensure its relevance and usability. Interactive activities are also useful for eliciting the children’s own narratives, fostering a deeper connection to the intervention.

## Discussion

In this participatory and pragmatic intervention design process, a previous scoping review provided a strong foundation of possible interventions for children that are hospitalised with TB. Various interventions involved children’s caregivers which improved the efficacy of the interventions [28–30]. In this study, the children are hospitalised with little to no contact with their caregivers or parents. Despite this, the intervention was feasible in a hospital setting. The incorporation of play into this intervention contributes to its success, which is consistent with the findings of various studies on the success of interventions which incorporate play for children who are hospitalised with cancer and other chronic illnesses [31,32].

This study shows that stakeholder engagement is integral in the development of storytelling interventions, as shown in a study describing the development of a storytelling intervention where teachers in low-income preschools were central to the development of the intervention [33].

During the development of a storytelling intervention in India, parents were trained to use culturally relevant interactive techniques during the intervention to ensure that the story was familiar to the children [34]. Similarly, in this study, feedback from healthcare workers shaped the adaptation of the story by making use of animals which were more familiar to the children.

The intervention toolkit, Courageous Curly, was implemented by the hospital schoolteacher. The teacher did not have any training with regards to storytelling and activities but managed to deliver the intervention successfully. This is unlike two of the studies found in the scoping review in which the authors used trained and certified individuals to present the interventions [31,35]. The teacher delivering the intervention did not have to be trained to facilitate the interactive activities; she was a hospital schoolteacher. This shows that an interactive storytelling intervention can be implemented successfully and the facilitator of the story does not require specific training on narrative interventions, even though the facilitator in the pilot was a qualified primary-school level educator. The prompts within the intervention were a useful guide for any storyteller. This indicates that the implementation of the story is feasible in a hospital setting such as the setting in which this study took place.

A study on a successful intervention in using storytelling as a tool to manage psychosocial distress during long-term hospitalisation [33] uses computers for the interventions. Although not all healthcare facilities that hospitalise children for TB would necessarily have access to computers, the school at the hospital that this study was conducted in does. This opens the possibility for the intervention to be digitalized for children at the hospital school.

The collaborative effort between the hospital staff, children and their parents/caregivers were useful in the development of this intervention as they have given insight into the challenges experienced by the children and the context in which the intervention would be implemented. This is consistent with the findings in a review that was conducted by Melnyk (2004) as it shows the usefulness of parental involvement in the psychological preparation of children for hospitalisation [36]. Parents and health workers should also continuously remind the children of why they are at the hospital and receiving medication as the children often forget.

## Limitations

The admission and discharge of children impose limits on extrapolation as the data reflects a specific, age-restricted, context-specific group meaning that the findings may not be generalisable beyond BCH or similar facilities. The use of convenience sampling resulted in a small sample of children being included in this study. The sample size was small and reflected the limited number of children at the hospital school who could meaningfully participate. There is also potential for observer bias during naturalistic observation, and the study did not measure psychosocial outcomes meaning that the intervention’s effectiveness remains unknown. The purpose of this pilot project was to develop the intervention. We acknowledge the need for further validation to establish its credibility, replicability, and effectiveness. We therefore recommend that future studies focus on validating the intervention.

## Conclusions

This study contributes to literature on how children’s negative experiences of hospitalisation can be managed. This is the first study on the development of an intervention for the management of psychosocial distress in children hospitalised with TB. This study demonstrated that gathering information from all stakeholders contributes to a feasible intervention for use in a hospital setting for children. Implementation and evaluation of such can inform interventions to mitigate the psychosocial impact of TB in children and inform policies to improve their overall TB care.
